# Two-sample mendelian randomization reveals a causal association between membranous nephropathy and lung cancer

**DOI:** 10.1038/s42003-023-05111-7

**Published:** 2023-09-01

**Authors:** Kezhen Yang, Xiaofeng Ding, Jipeng Liu, Saisai Liu, Qingguo Liu, Jianhua Li, Pingna Zhang

**Affiliations:** 1 Department of Rehabilitation Medicine, Sir Run Run Shaw Hospital, Zhejiang University School of Medicine, 3 East Qingchun Road, Hangzhou, Zhejiang 310016 China; 2 Shanghai Skin Disease Hospital, School of Medicine, Tongji University, Shanghai, China; 3 School of Acupuncture-Moxibustion and Tuina, Beijing University of Chinese Medicine, Beijing, 102488 China; 4 China-Japan Friendship Hospital, Beijing, China; 5 Department of Nephrology, The First Affiliated Hospital of Zhejiang Chinese Medical University (Zhejiang Provincial Hospital of Chinese Medicine), Hangzhou, China

**Keywords:** Lung cancer, Kidney diseases

## Abstract

A risk association between membranous nephropathy (MN) and lung cancer is reported, but traditional observational studies cannot provide strong evidence of its causality. This study aimed to assess genome-wide association studies data for a causal relationship between MN and lung cancer using a two-sample Mendelian randomization (MR) approach. Inverse-variance weighted, and MR Egger regression techniques were used to determine the association of genetic variants from cohorts of MN and lung cancer patients. Independent genetic variants with genome-wide significance (*P* < 5×10^–8^) were used to determine the direction of chance. Sensitivity analyses confirmed the accuracy of the results. The results suggest that MN is an exposure factor for lung cancer, validated using a second cohort of lung cancer patients (*P* < 0.001). There is insufficient evidence to suggest a causal relationship between lung cancer and MN; however, cigarette smoking may be a confounding factor for lung cancer due to MN. The findings provide causal evidence for the effect of MN on lung cancer risk and may be useful for patient management, especially in older patients with MN who should be systematically screened regularly.

## Abstract (web summary)

A two-sample Mendelian randomization study identifies a causal effect of membranous nephropathy on lung cancer, suggesting that membranous nephropathy may be an exposure factor for lung cancer.

## Introduction

Membranous nephropathy (MN) is a distinct glomerular lesion associated with other systemic diseases or exposures (secondary MN) in approximately 20% of patients^1^. With the gradual discovery of in situ antigens, the majority of previous investigations have concentrated on examining the variations in pathophysiology between primary (idiopathic) and secondary (cancer-associated) MN and exploring the differences in pathogenesis between idiopathic and secondary MN. Since Lee^2^ first proposed a connection between nephrotic syndrome and cancer in 1966, scientists have been identifying ways in which these diseases are related. Clinically, cancer-related nephropathy is on the rise, but the pathological types are atypical and the etiology is unclear^3,4^. Although several reports mention improvement in symptoms of nephrotic syndrome in patients with MN after aggressive tumor treatment^5–7^, the evidence that directly reveals this relationship is still limited, controversial, and speculative^8^. It has been reported that the processes of MN and malignancy often develop in parallel, with tumor antigens or tumor-reactive antibodies found within glomerular immune deposits, supporting an association between the two diseases^9^. However, both MN and malignancy are prone to occur in middle-aged and elderly populations, so the presence of both diseases in the same patient may also be coincidental. Thus, a causal relationship between tumor and MN has not been universally accepted, and whether malignancy-associated MN exists independently of the concept of secondary MN as a distinct entity deserves further investigation.

Several studies have reported an association between MN and cancer^7,10,11^, with lung cancer having the strongest correlation. Lung cancer may occur after the diagnosis of MN^11^, but there are few studies on whether MN leads to an increased risk of lung cancer, and on the mechanisms of association between these diseases. In observational studies, bias due to confounding and reverse causality cannot be completely excluded, so the specific causal relationship between MN and lung cancer is unclear. Therefore, we performed a two-sample bidirectional Mendelian randomization (MR) analysis to investigate the causal effect between MN and lung cancer.

## Results

### MR analysis results

**Fig. 1:**
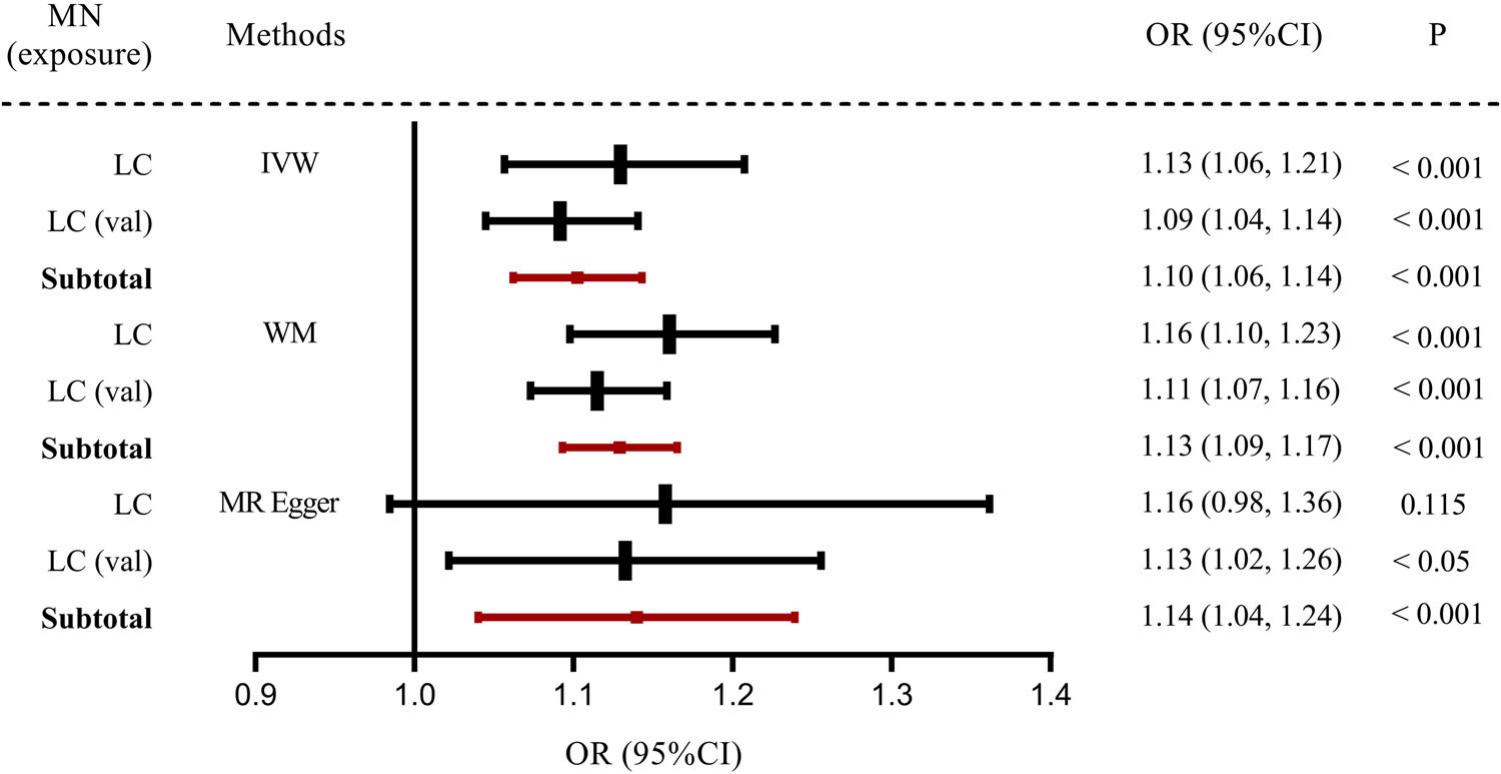
Forest plot of the association of MN with lung cancer. Different methods were used to obtain OR values and 95% confidence intervals. Subtotal estimates were combined using a fixed effects meta-analysis and the results are shown in red. Subtotal indicates the summary of the analysis results for different datasets. MN membranous nephropathy, LC lung cancer, CI confidence interval, OR odds ratio, IVW inverse-variance weighted, WM weighted median.

First, we analyzed whether MN was the cause of lung cancer; the results are shown in Fig. 1. A total of 10 SNPs were identified in the lung cancer cohort. The IVW and WM models showed statistically significant estimates of the effect of MN on lung cancer (*P* < 0.001); the MR Egger model yielded a *P* = 0.115, which was not significantly different, but consistent in direction with the other models. Ten SNPs were identified in the lung cancer validation cohort, and the results of all three models were statistically significant (*P* < 0.001). The results of the validation cohort support the results of the exploration cohort, suggesting a causal relationship between MN and lung cancer, and that MN may lead to lung cancer.

The reverse causality between MN and lung cancer was analyzed to determine if lung cancer was the cause of MN. The calculated results for the IVW, WM, and MR Egger models are shown in Table 1. The lung cancer and lung cancer validation cohorts, gave a *P* > 0.05 for all three models, indicating that lung cancer was not the cause of MN. However, owing to the small number of identified SNPs, this result cannot fully represent the causal relationship between lung cancer and MN.

**Table 1:** MR analysis results of lung cancer with MN.

Exposure	nSNP	Methods	*P*
Lung cancer	5	IVW	0.413266
		WM	0.2245805
		MR Egger	0.8555143
Lung cancer (val)	3	IVW	0.3260229
		WM	0.1013952
		MR Egger	0.3886883

*MN* membranous nephropathy, *val* validation, *SNP* single-nucleotide polymorphisms, *IVW* inverse-variance weighted, *WM* weighted median.

### Sensitivity analyses results

**Fig. 2:**
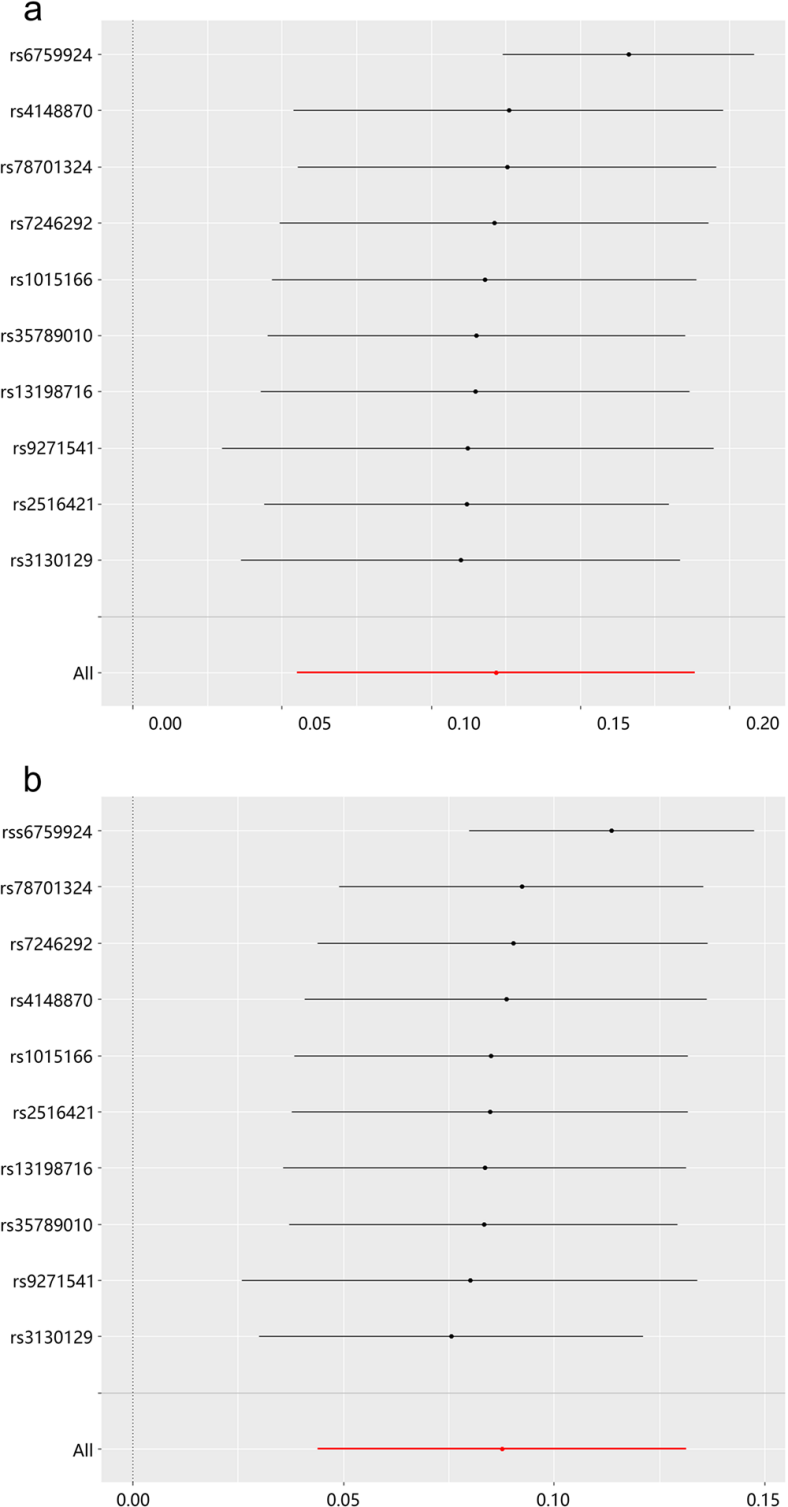
Leave-one-out plots for the causal effect of MN on lung cancer. **a** MN on lung cancer. **b** MN on lung cancer (val). The black line is the deviation of the 95% confidence interval corresponding to the estimate of the SNPs. The red line corresponds to the estimated value of the IVW test. After removing SNPs one by one, there was no difference with the final result. MN membranous nephropathy, val validation, SNPs single-nucleotide polymorphisms.

After completing the MR analysis, sensitivity analysis was performed on the results of the MR analysis. The results of the leave-one-out sensitivity test for the causal analysis of MN and lung cancer are shown in Fig. 2. The results of the lung cancer and lung cancer validation cohorts are shown in Fig. 2a, b respectively. This shows that the results of the MR analysis are plausible.

Heterogeneity testing was performed using Cochran’s *Q* test (Table 2), which yielded *P* < 0.05 for both the IVW and MR Egger models. This indicated that heterogeneity existed and that a causal relationship between MN and lung cancer was not due to sampling error. This was further confirmed by calculating the *P* value of the IVW analysis in the random effects model (Table 2). Thus, the occurrence of MN may lead to an increased risk of developing lung cancer.

**Table 2:** Cochran’s *Q* test.

Outcome	Method	*Q*	*P*	BETA	SE	IVW-P
Lung cancer	MR Egger	28.06881	0.000461	0.1217668	0.03402443	0.0003451682
	IVW	28.45184	0.000801			
Lung cancer (val)	MR Egger	28.50065	0.000388	0.08764764	0.0223608	0.00008865926
	IVW	30.65122	0.00034			

*val* validation, *IVW* inverse-variance weighted, *BETA* effect size for the effect allele, *SE* standard error.

*P* > 0.05 indicates no heterogeneity.

The results of the pleiotropic tests are presented in Table 3. In both lung cancer cohorts, there was no directional pleiotropy (*P* > 0.05). The results of the above sensitivity analysis support the results of the MR analysis for a causal relationship between MN and lung cancer. Scatter plots, forest plots, and funnel plots of SNPs are shown in Supplementary Fig. S1–3.

**Table 3:** Pleiotropy test by MR Egger intercept.

Outcome	Intercept	*P*
Lung cancer	-0.017162473	0.749580158
Lung cancer (val)	-0.025946944	0.459542094

*val* validation.

*P* > 0.05 indicates no horizontal polymorphism.

**Fig. 3:**
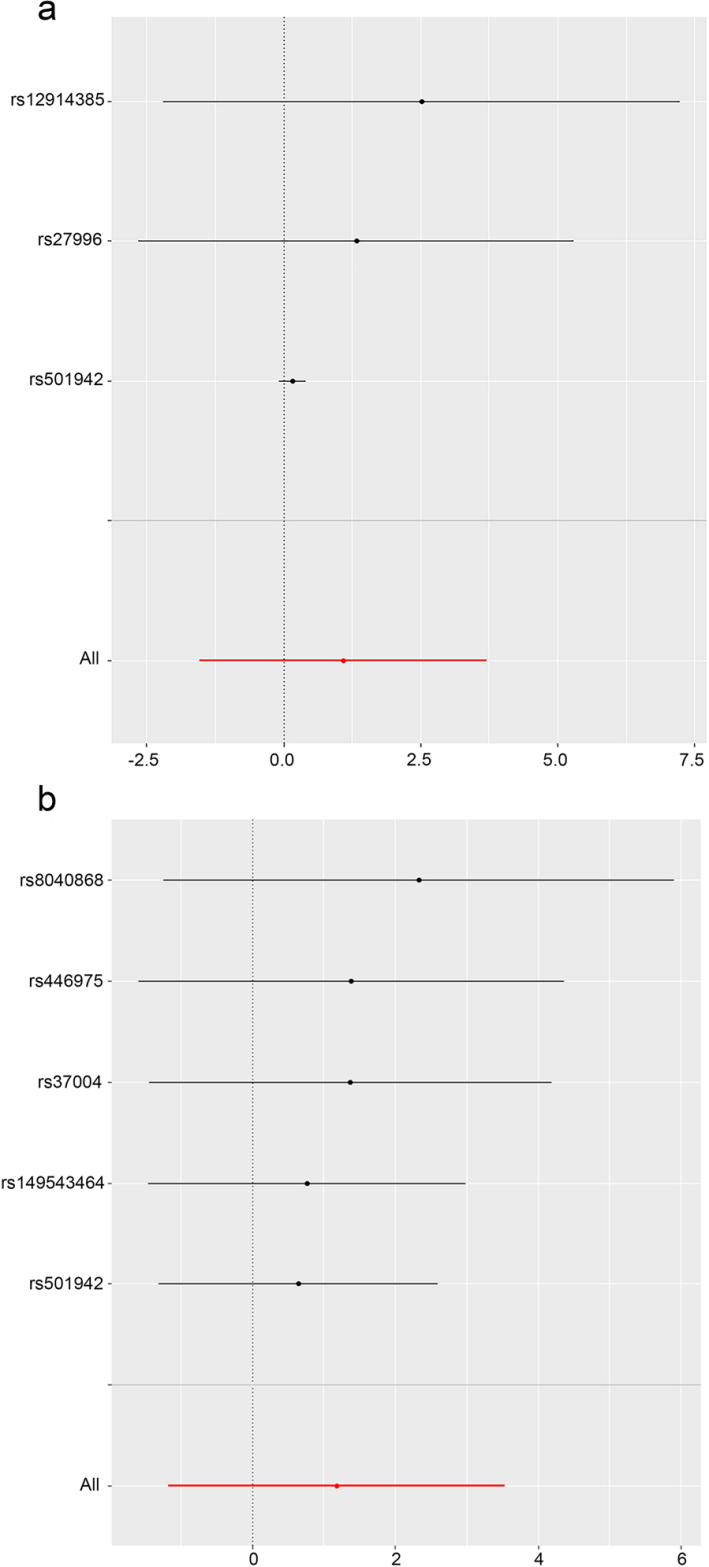
Leave-one-out plots for the causal effect of lung cancer on MN. **a** Lung cancer with MN. **b** Lung cancer (val) with MN. The black line is the deviation of the 95% confidence interval corresponding to the estimate of the SNPs. The red line corresponds to the estimated value of the IVW test. After removing SNPs one by one, there was no difference with the final result. MN membranous nephropathy, val validation, SNPs single-nucleotide polymorphisms.

The results of the sensitivity analysis of the causal relationship between lung cancer and MN are shown in Fig. 3 and Table 4. High heterogeneity was observed in the results of the MR analysis with no directional pleiotropy. The related visualization results are shown in Fig. S4–6. In the MR analysis, the number of SNPs obtained was low, and on balance, the result that there is no causal relationship between lung cancer and MN has yet to be verified.

**Table 4:** Sensitivity analyses (lung cancer with MN).

Heterogeneity test				Pleiotropy test		
Exposure	Method	*Q*	*P*	Exposure	Intercept	*P*
Lung cancer	MR Egger	343.3515	4.10E-74	Lung cancer	1.413723	0.278838
	IVW	542.4417	4.42E-116			
Lung cancer (val)	MR Egger	285.0128	6.07E-64	Lung cancer (val)	-0.15822	0.944825
	IVW	287.1644	4.40E-63			

*val* validation, *IVW* inverse-variance weighted.

### Effect of confounding factors

Considering the effect of confounding factors on the outcome variables, smoking, prostate cancer, and colorectal cancer were included as exposure factors along with MN in the multivariate MR analysis. The causal relationship between each variable and lung cancer were analyzed using the IVW model. Information on the selected dataset and the results of the MR analysis are shown in Table 5. Only MN and smoking had a *P* < 0.05, indicating that when smoking and MN occur together, they may contribute to the development of lung cancer. Prostate cancer and colorectal cancer, on the other hand, would not be influential factors in the causal relationship between MN and lung cancer.

**Table 5:** Analysis of confounding factors.

No	GWAS ID	Confounding factors	nSNP	BETA	SE	*P*
1	ebi-a-GCST010005	MN	3	0.067415	0.02799	0.016018
2	ieu-b-4965	Colorectal cancer	5	6.665933	6.708721	0.320407
3	ukb-a-16	Smoking	12	3.538611	0.995549	0.000379
4	ukb-b-13348	Prostate cancer	12	-8.39319	7.008562	0.231087

*GWAS* genome-wide association study, *SNP* single-nucleotide polymorphism, *BETA* effect size for the effect allele, *SE* standard error.

## Discussion

The present MR study assessed the association between MN and lung cancer from a genetic perspective. The results of the exploratory cohort study supported a causal relationship between MN and lung cancer. MN may be an exposure factor that causes lung cancer, and the results of the validation cohort were identical. Many studies have identified an association between MN and lung cancer; however, the exact causality and pathogenesis of this association remain unclear.

Epidemiological evidence has shown that the incidence of cancer is significantly higher in patients with MN than in the general population. After MN is diagnosed in patients, the risk of developing cancer may persist for at least five years^3^, and the risk of MN-related cancers increases with patient age^12,13^. Meanwhile, in a meta-analysis^11^, the incidence of cancer-associated MN was found to be approximately 10%, with lung cancer being the most common, followed by stomach, bowel, prostate, and breast cancers. A small number of patients with lung cancer may present with clinical manifestations led by nephropathy, which resolves after treatment of lung cancer^4^, and in patients with cancer-associated MN, there is a strong association between reduced proteinuria and remission of clinical symptoms of cancer^14^. In a recent study, six patients with lung cancer and nephropathy as the first manifestation were followed up and analyzed. Most of these patients did not show clinical manifestations or specific tumor markers associated with lung cancer, but bilateral lower limb edema associated with MN was the main manifestation^4^. Our MR analysis revealed that MN may act as an exposure factor for lung cancer, which is consistent with the clinical presentation of symptoms. However, the malignancy has a long latency period, and although MN is the first symptom diagnosed in the clinic, the causal relationship between the two still requires further investigation. Therefore, tumor marker screening, with chest CT screening if required, should be performed as early as possible to determine the presence of cancer in elderly patients with MN and not rely solely on its clinical manifestations. In the follow-up of elderly patients with MN who are not in remission with conventional treatment, regular systemic examinations should be performed. Furthermore, cigarette smoking is the most important risk factor for lung cancer in both men and women^15^. In our study, the confounding factor analysis indicated that smoking may be a confounding factor when MN causes lung cancer, which is consistent with previous findings. Thus, lung cancer should be systematically investigated in patients with MN who are smokers.

The M-type phospholipase A2 receptor (PLA2R) is identified in 70–80% of idiopathic MN^16^. Furthermore, it has been found that the absence of glomerular PLA2R and the dominance of IgG1- and/or IgG2-restricted subclasses are common in patients with malignancy-associated MN^17^. Furthermore, IgG4 is the major IgG subclass in primary model glomerular immune deposits. It has been found that IgG4 deficiency in glomeruli may cause malignancy, but this has not yet been confirmed^17–19^, whereas IgG1 and IgG2 deposition were associated with malignancy^19^, with a predominance of Th1-type responses in cancer-associated MN. Thrombospondin type-1 domain-containing 7 A (THSD7A) is a MN autoantigen and a secondary autoantigen of MN^20^, which has been found to be associated with the development of malignant tumors, with one study showing that THSD7A positivity in malignant tumors is 15–20%^21^. A possible molecular link between increased THSD7A expression in tumors and the presence of positive THSD7A antibodies in MN has been reported, suggesting a similar pathogenesis between primary and malignancy-associated MN^22^. In a clinical study, positive THSD7A staining was found in both glomeruli and cancer cells of patients with lung cancer, and remission of MN symptoms was observed after surgical resection, confirming the association of THSD7A with lung cancer and MN^23^. Recently, neural epidermal growth factor-like 1 (NELL1) was identified as a new antigen in 3.8% of primary MN^24^. Compared to PLA2R- and THSD7A-associated cases of MN, NELL1-positive MN has a higher proportion of cases with malignancies (33%)^25^.

Distinguishing between idiopathic MN coexisting with malignancy and malignancy-associated MN is difficult, especially in elderly patients where the occurrence of malignancy is common. This study aimed to extend previous research by demonstrating a causal relationship between MN and lung cancer. Although the pathophysiological mechanism underlying this relationship is still unclear, there is no doubt that there are some common biological pathways between them. Evidence indicates that THSD7A is a potential tumor antigen in humans, participating in cancer progression, vascular invasion, angiogenesis, and metastasis in the tumor environment^26–28^. Additionally, THSD7A was detected in lung cancer tissue, which was also THSD7A-positive MN^23^. A possible explanation is that the immune system forms an anti-THSD7A antibody that also attracts antibodies against THSD7A antigens in cancer cells. Circulating immune complexes formed by the shedding of tumor antigens are trapped in the glomerular capillary wall, causing an immune response. Tumor initiation is a process in which normal cells acquire the first mutational hit, and inflammatory microenvironments can contribute to mutation rates^29^. In patients with MN and malignancy, renal biopsy results suggest a significantly higher number of inflammatory cells infiltrating the glomeruli^14^. We speculate that activated inflammatory cells can induce DNA damage and genomic instability, which are involved in tumorigenesis. Furthermore, the study found that PLA2R1 and THSD7A were expressed not only in podocytes but also in pulmonary fine bronchi^22^, which may also be a potential cause of lung cancer in MN. The lungs have a large surface area exposed to the outside world. Extrinsic processes, such as viral infections or potentially abnormal immune responses, may cause MN in cancer^30^.

To our knowledge, the present study is the first to use a two-sample MR analysis to examine the causal relationship between MN and lung cancer. Compared with observational studies, MR analysis is less prone to confounding factors, antinomial causality, and errors caused by non-differential measurements^31^. The results of multiple sensitivity analyses supported the MR analysis conclusion that the findings were not confounded by polymorphic factors. However, the reverse causality between MN and lung cancer could not be effectively confirmed, owing to the quality of the GWAS dataset. The total sample size of the MN dataset was small, which may have led to errors. The lung cancer dataset lacks a classification of different lung cancer types; thus, the association between lung cancer types and MN could be further explored. The GWAS dataset is European in ethnicity, and lacks data on other ethnicities, and thus has limited generalizability to other populations.

In conclusion, our study provides genetic evidence that there is a causal relationship between MN and lung cancer and that MN may be a cause of lung cancer. This suggests that awareness of the potential risk of developing lung cancer in patients with MN needs to be raised in the clinic, which could help in early disease intervention. Exploring the specific biological mechanisms that induce lung cancer in patients with MN should be the subject of further research.

## Methods

### Study design

A two-sample MR analysis between MN and lung cancer, utilizing summary statistics from genome-wide association studies (GWAS), was performed to evaluate their bidirectional causal relationship and were validated using multiple datasets.

### Study samples and measures

According to Mendel’s law of inheritance, during meiosis, chromosomes carry genes that are randomly assigned to offspring without confounding factors, allowing the simulation of randomized controlled experiments. Here, we obtained genetic variables between MN and lung cancer using GWAS.

The details of each cohort are presented in Table 6. The MN cohort (ebi-a-GCST010005) contained 7979 samples with 5,327,688 SNPs obtained^32^. All subjects were primary MN patients. And all subjects in this cohort were genotyped using high-density SNP arrays, and approximately 7 million common, high-quality markers were imputed using the latest genome-wide sequence reference panel. The lung cancer exploration cohort (ieu-a-967) contained 18,313 samples, and the lung cancer validation cohort (ieu-a-966) contained 27,209 samples^33^. In both lung cancer cohorts, all subjects underwent standard quality control; individuals with low call rates and very high or very low heterozygosity as well as individuals of non-European ancestry, were excluded. To ensure the quality of genotyping in all tests, duplicate samples were genotyped at each center. To exclude technical errors, cross-platform validation was performed on some samples to ensure genotyping accuracy.

**Table 6:** Characteristics of data. The disease datasets are all from the IEU OpenGWAS project (https://gwas.mrcieu.ac.uk/).

Characteristic	GWAS ID	Population	Controls	Cases	Number of SNPs
MN	ebi-a-GCST010005^32^	European	5829	2150	5327688
Lung cancer	ieu-a-967	European	15038	3275	8893750
Lung cancer (val)	ieu-a-966^33^	European	15861	11348	8945893

*MN* membranous nephropathy, *val* validation, *SNPs* single-nucleotide polymorphisms, *GWAS* genome-wide association study.

Ethical approval and consent to participate were obtained from the original publication. Informed written consent was obtained from all participants in the MN and lung cancer cohorts All studies were reviewed and approved by the ethics review boards of the relevant institutions.

### MR analysis

For analysis using the TwoSampleMR package, the criteria for identifying genetic variants were kb = 1000, r^2^ = 0.01. The specific information on MN as an exposure factor for identifying SNPs is shown in Table 7. *F*-statistics is a common metric for assessing bias in weak instrumental variables, *F*-statistics = (β/SE)^2^ ^34^. The mean value of *F*-statistics for all SNPs was 133.6589. *F*_max_ = 471.5235, *F*_min_ = 30.80498. The *F*-statistics > 10 for each SNP indicates that there is no bias due to weak instrumental variables^35^. There will be no impact on the results of MR analysis. The results of lung cancer as an exposure factor for identifying SNPs are shown in Table S1–2. In the process of identifying instrument variables (IVs), no data coordination was performed. The causal relationship between MN and lung cancer was confirmed using the inverse-variance weighted (IVW) model combined with the weighted median (WM) and MR Egger models.

**Table 7:** Explore cohort SNPs.

No	SNP	Effect allele	Other allele	*P*	β	SE	*F*
1	rs6759924	A	G	1.98E-48	-0.6906	0.0472	214.0766
2	rs78701324	G	A	2.37E-09	0.7207	0.1207	35.65287
3	rs28732209	C	A	2.10E-08	-0.6025	0.1075	31.41211
4	rs9271541	C	A	2.19E-104	1.0119	0.0466	471.5235
5	rs1131114	C	T	4.42E-29	0.5136	0.0459	125.2059
6	rs2516421	T	C	5.19E-09	-0.594	0.1017	34.11387
7	rs4148870	T	C	3.69E-17	0.3561	0.0423	70.87013
8	rs13198716	T	C	8.56E-35	0.8988	0.073	151.5934
9	rs9257809	G	A	1.32E-45	0.9578	0.0676	200.7503
10	rs9275518	A	G	7.50E-31	-0.4924	0.0426	133.6032
11	rs35789010	A	G	4.83E-22	0.8001	0.0829	93.14928
12	rs3132473	A	T	8.26E-92	1.2784	0.0629	413.0782
13	rs3094673	T	C	1.16E-08	-0.4945	0.0867	32.53077
14	rs2524236	G	C	3.39E-10	-0.3622	0.0577	39.40445
15	rs1015166	T	C	2.34E-15	0.3446	0.0435	62.75553
16	rs3130129	C	T	6.14E-62	1.0826	0.0652	275.7026
17	rs111876947	G	A	2.87E-08	0.4973	0.0896	30.80498
18	rs72854513	T	A	1.23E-40	0.9365	0.0702	177.9678
19	rs3135024	C	T	1.07E-08	-0.2946	0.0515	32.72284
20	rs7246292	C	T	1.09E-11	0.2775	0.0408	46.26

*SNP* single-nucleotide polymorphisms *β* effect size for the effect allele *SE* standard error. *F* F-statistics.

### Sensitivity analysis

Sensitivity analysis included the leave-one-out sensitivity, heterogeneity, and pleiotropy tests. The leave-one-out sensitivity test calculates the MR results of the remaining IVs after eliminating the IVs individually. If the calculated results of the other IVs are significantly different from the final results after the elimination of a certain IV, then the MR results are sensitive to that IV. The heterogeneity test calculates the differences between individual IVs; the greater the differences between different IVs, the greater the heterogeneity of these IVs^36^. The pleiotropy test checks for directional pleiotropy between different IVs, expressed by the intercept of the MR Egger regression; if the intercept is very different from 0, it indicates the presence of a horizontal multi-effect^37^. All statistical tests were performed between two samples using the Two-Sample MR (version 0.5.6) package of the R software (Version 4.2.0).

### Analysis of confounding factors

Considering complex biological factors, multivariate MR analysis was used to exclude possible confounding factors. Previous studies have found that the vast majority of tumors associated with MN are lung, prostate, and colorectal cancers^11^. In addition, an association between smoking^38^, MN, and lung cancer has been reported. In the confounding factor analysis, MN, smoking, prostate cancer, and colorectal cancer were considered exposure factors, and the causal relationship between them and lung cancer was analyzed to determine what factor had on the estimate of effect.

### Statistics and reproducibility

In this study, two-sample MR analysis was used to explore the causal relationship between MN and lung cancer. Comprehensive information on SNPs, particularly effector alleles, was also included. The main MR analysis methods used were IVW, WM, and MR Egger. The risk of MN causing changes in lung cancer was expressed using odds ratios (ORs) and 95% confidence intervals (CI). *P* < 0.05 indicates a statistical difference.

### Reporting summary

Further information on research design is available in the Nature Portfolio Reporting Summary linked to this article.

### Supplementary information

Supplementary Figures and Tables

Description of Additional Supplementary Files

Supplementary Software 1

Supplementary Data 1

Reporting Summary

## Data Availability

All the datasets we used are freely available from IEU OpenGWAS project (https://gwas.mrcieu.ac.uk/). The data used for the analysis can be found in the “Supplementary Data 1”.

## Code availability

The code used in this study can be found in "Supplementary Software 1".
