# Supplementary material for: Two-sample mendelian randomization reveals a causal association between membranous nephropathy and lung cancer
Source: Commun Biol. 2023 Sep 1;6:887. doi: 10.1038/s42003-023-05111-7 (PMC10474265; doi:10.1038/s42003-023-05111-7)
Supplement: Supplementary file 1 — Supplementary Figures and Tables [file 42003_2023_5111_MOESM1_ESM.pdf]

Supplementary Material

No	SNP	Effect allele	Other allele	P	BETA	SE
1	rs27996	G	A	1.84E-08	-0.16581	0.024244
2	rs501942	T	C	6.74E-09	0.262723	0.056353
3	rs11571818	C	T	6.28E-09	0.866457	0.307405
4	rs12914385	T	C	2.30E-25	0.299604	0.037745

Table S1 Explore cohort SNPs (LC). SNP, single-nucleotide polymorphisms. BETA, effect size for the effect allele. SE, standard error.

No	SNP	Effect allele	Other allele	P	BETA	SE
1	rs446975	T	G	6.42E-18	-0.26153	0.022649
2	rs37004	T	C	3.19E-13	-0.17209	0.019417
3	rs501942	T	C	1.47E-10	0.186841	0.034137
4	rs149543464	A	G	1.07E-08	0.168276	0.033808
5	rs11571818	C	T	2.63E-12	0.660847	0.16686
6	rs8040868	C	T	4.97E-60	0.301974	0.024535

Table S2 Explore cohort SNPs (Lung cancer-val). SNP, single-nucleotide polymorphisms. BETA, effect size for the effect allele. SE, standard error.

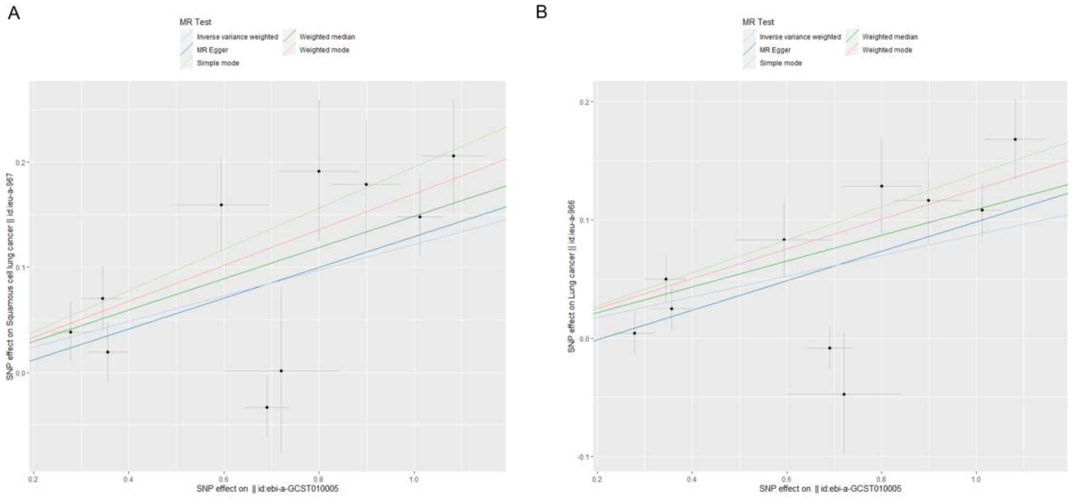

Figure S1 Scatter plot to visualize causal effect of MN on the risk of lung cancer. The slope of the straight line indicates the magnitude of the causal association. (A) Lung cancer. (B) Lung cancer (val). MN, membranous nephropathy. val, validation.

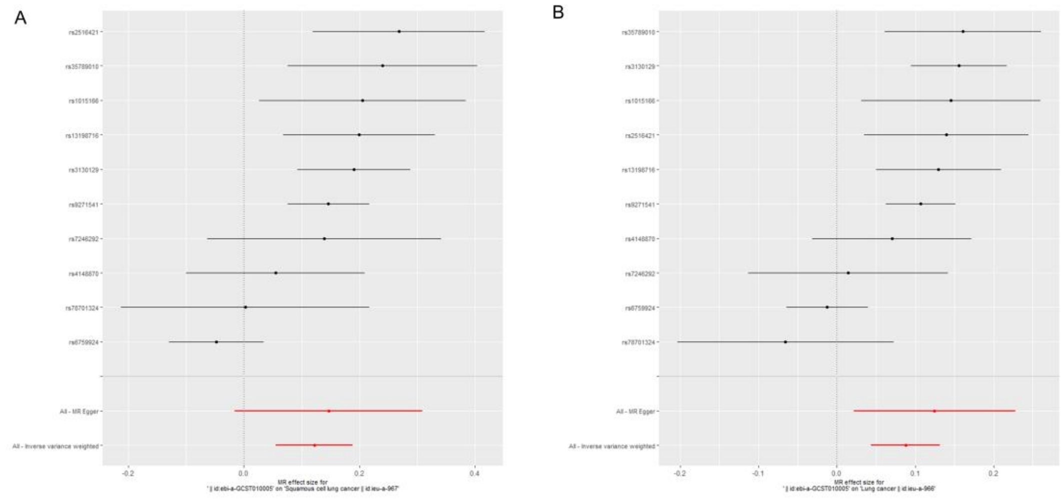

Figure S2 Forest plot to visualize causal effect of MN on lung cancer. (A) Lung cancer. (B) Lung cancer (val). MN, membranous nephropathy. val, validation.

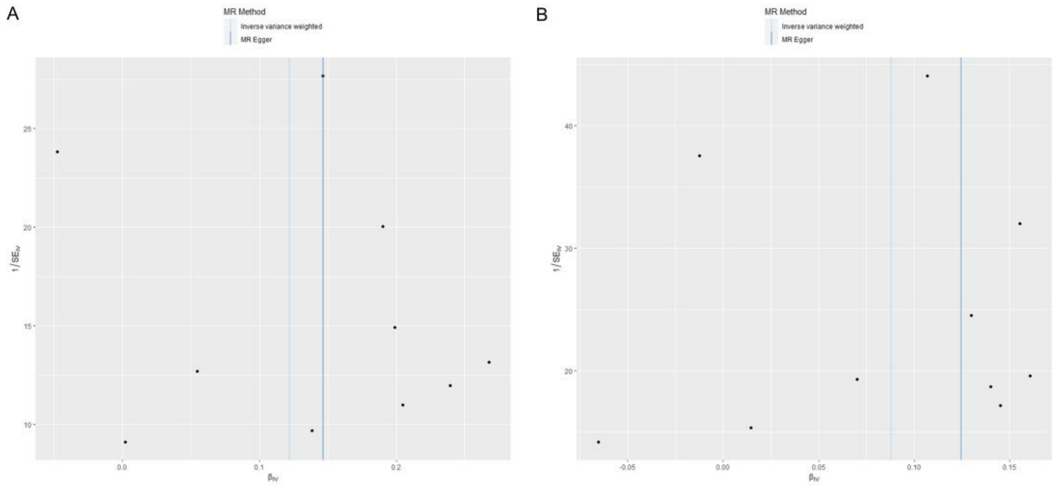

Figure S3 Funnel plots to visualize overall heterogeneity of MR estimates for the effect of MN on lung cancer. (A) Lung cancer. (B) Lung cancer (val). MN, membranous nephropathy. val, validation.

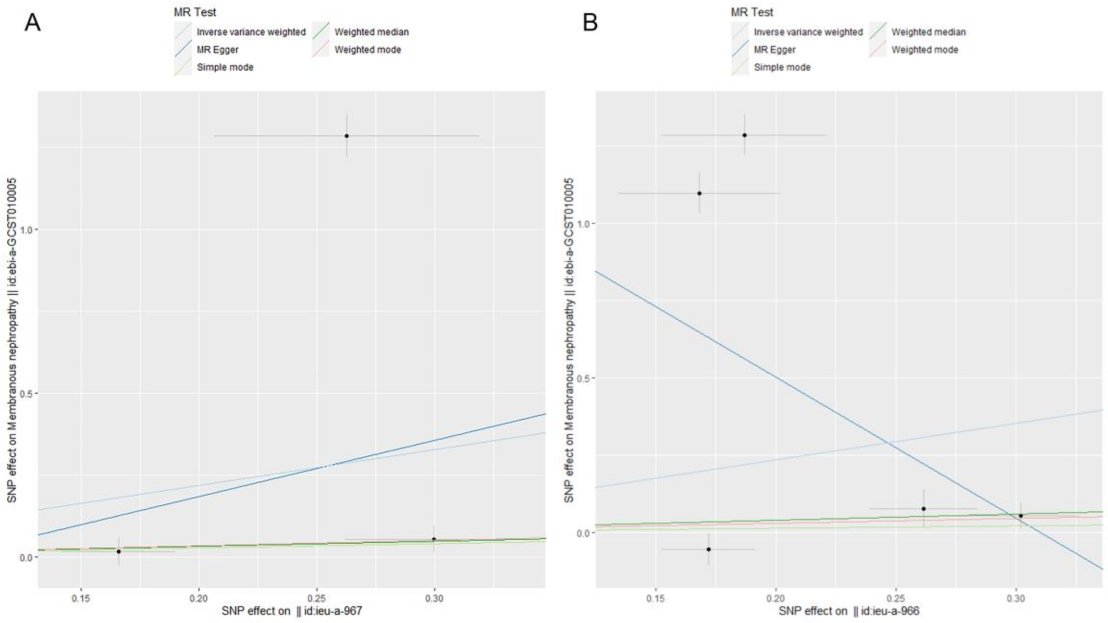

Figure S4 Scatter plot to visualize causal effect of lung cancer on MN. The slope of the straight line indicates the magnitude of the causal association. (A) Lung cancer. (B) Lung cancer (val). MN, membranous nephropathy. val, validation.

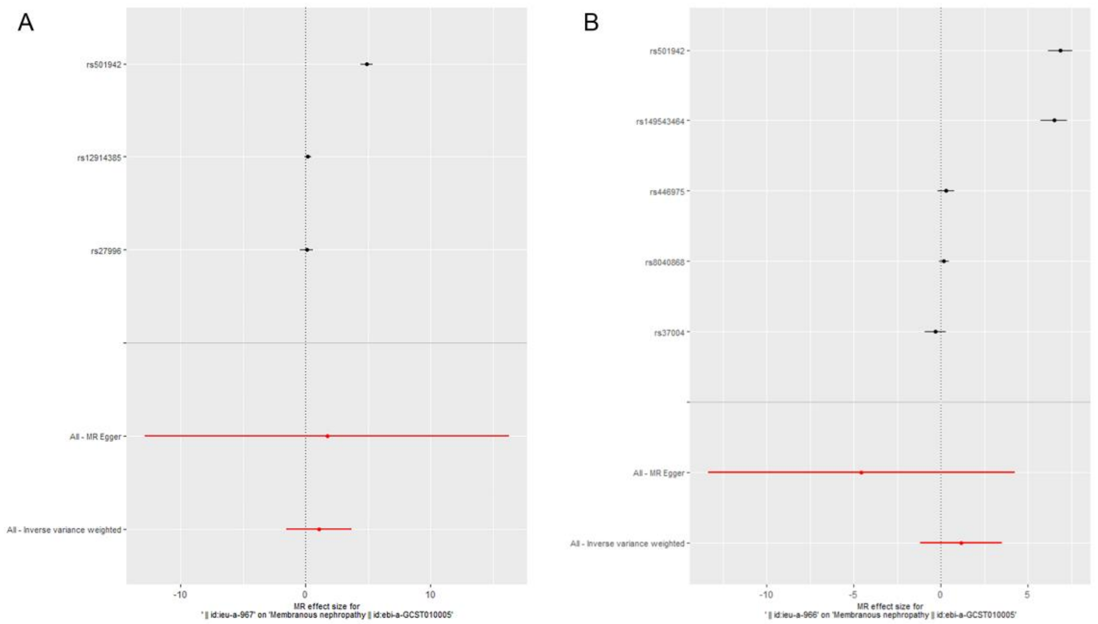

Figure S5 Forest plot to visualize causal effect of lung cancer on MN. (A) Lung cancer. (B) Lung cancer (val). MN, membranous nephropathy. val, validation.

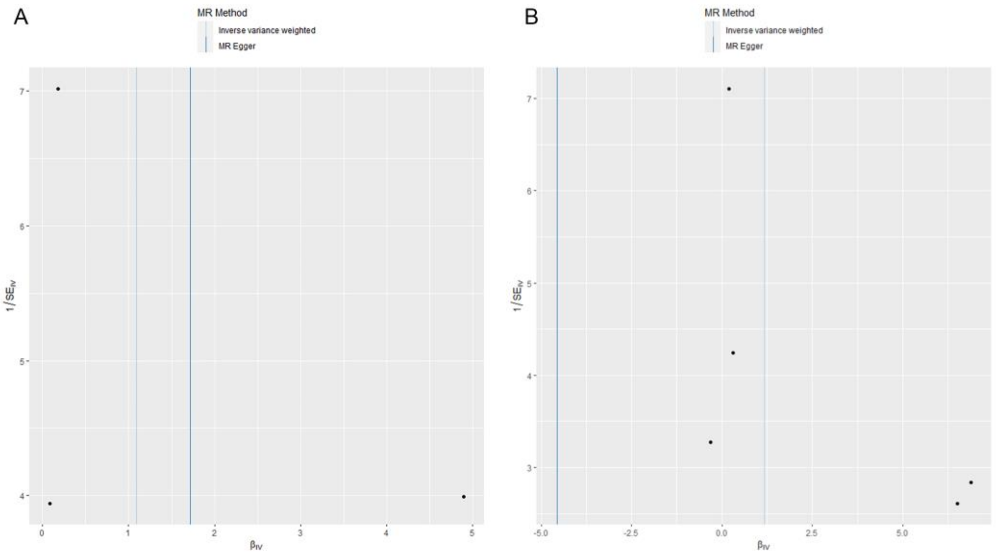

Figure S6 Funnel plots to visualize overall heterogeneity of MR estimates for the effect of lung cancer on MN. (A) Lung cancer. (B) Lung cancer (val). MN, membranous nephropathy. val, validation.
