# Supplementary material for: Two-sample mendelian randomization reveals a causal association between membranous nephropathy and lung cancer
Source: Commun Biol. 2023 Sep 1;6:887. doi: 10.1038/s42003-023-05111-7 (PMC10474265; doi:10.1038/s42003-023-05111-7)
Supplement: Supplementary file 2 — Description of Additional Supplementary Files [file 42003_2023_5111_MOESM2_ESM.pdf]

Description of Additional Supplementary Files

File name: Supplementary Data 1
Description: The source data behind the Fig 1-3 in the paper.

File name: Supplementary Software 1
Description: The code used in this study.
