# Supplementary material for: Two-sample mendelian randomization reveals a causal association between membranous nephropathy and lung cancer
Source: Commun Biol. 2023 Sep 1;6:887. doi: 10.1038/s42003-023-05111-7 (PMC10474265; doi:10.1038/s42003-023-05111-7)
Supplement: Supplementary file 3 — Supplementary Software 1 [file 42003_2023_5111_MOESM3_ESM.pdf]

```
#install.packages('devtools')
#devtools::install_github('MRCIEU/TwoSampleMR')

library(TwoSampleMR)

#MR

#exposure,kb=1000 到 10000，r2=0.01 到 0.001

exposure_dat <-extract_instruments(outcomes="ebi-a-GCST010005",
                                   clump=T, r2=0.01,kb=1000,access_token = NULL)
#outcome
outcome_dat    <-extract_outcome_data(snps=exposure_dat$SNP,    outcomes="ieu-a-967")

dat <- harmonise_data( exposure_dat = exposure_dat, outcome_dat = outcome_dat )

res <- mr(dat)
res

#
#Leave-one-out
res_loo <- mr_leaveoneout(dat)
mr_leaveoneout_plot(res_loo)

#Heterogeneity test
Q<-mr_heterogeneity(dat)
Q

#Pleiotropy test
Pleiotropy<-mr_pleiotropy_test(dat)
Pleiotropy


#Visualization
mr_scatter_plot(res, dat)

res_single <- mr_singlesnp(dat)
mr_forest_plot(res_single)

mr_funnel_plot(res_single)
#IF P>0.05, next
#mr(dat,method_list = c('mr_ivw_mre'))

#Multi-factor confounding analysis
```

```
id_exposure <- c("ebi-a-GCST010005","ukb-a-16","ukb-b-13348","ieu-b-4965")
id_outcome <- "ieu-a-967"
exposure_dat <- mv_extract_exposures(id_exposure)
dim(exposure_dat)
#View(exposure_dat)

outcome_dat <- extract_outcome_data(exposure_dat$SNP, id_outcome)
mvdat <- mv_harmonise_data(exposure_dat, outcome_dat)
res <- mv_multiple(mvdat)
res
```
